# Bullous congenital diffuse cutaneous mastocytosis^☆^^☆☆^

**DOI:** 10.1016/j.abd.2019.01.012

**Published:** 2020-01-31

**Authors:** Julia Marcon Cardoso, Camila Angelico S. Cabral, Rute Facchini Lellis, Flavia Naranjo Ravelli

**Affiliations:** a Department of Dermatology, Universidade de Santo Amaro, São Paulo, SP, Brazil; b Sector of Dermatology, Department of Pathology, Hospital Santa Casa de Misericórdia de São Paulo, São Paulo, SP, Brazil; c Department of Dermatology, Hospital e Maternidade Santa Joana, São Paulo, SP, Brazil

Dear Editor,

Mastocytosis is a heterogeneous group of disorders whose signs and symptoms are characterized by mast cells infiltrate in tissues.^1^, ^2^ There are two main variants, cutaneous mastocytosis (CM) that is limited to the skin, and systemic mastocytosis (SM) in which extracutaneous organs are affected, such as the bone marrow, liver, spleen and lymphoid tissue.^1^, ^2^, ^3^ The skin is the most commonly affected organ, and in children it is often the only one. Studies suggest that in 15–31% of all patients the disease is congenital.^3^ Mastocytosis is also classified into two groups according to the age of onset: childhood and adult mastocytosis. The adult mastocytosis tends to progress to SM with worse prognosis, whereas childhood mastocytosis rarely progresses to a SM and tends to improve during adolescence.^1^, ^4^ Diffuse cutaneous mastocytosis (DCM) is a rare and severe variant that typically presents in the neonatal period with a sontroversial prognosis.^3^

A full-term male newborn, with no significant family or gestational history, presented at birth diffuse erythematous leathery plaques distributed throughout the body (Fig. 1). Physical examination did not present alterations in other organs or systems. On the third day of life, there were tense bullae (filled with yellowish fluid)) in the cephalic segment, upper and lower limbs and anterior trunk. In addition, the erythematous plaques became brownish, with *peau d’orange* appearance (Fig. 2). The spread of new bullous lesions was related to the heat of the incubator, handling with latex gloves and contact with clorexidine during hygiene. Laboratory and imaging investigations did not show any evidence of abnormalities.

**Figure 1:**
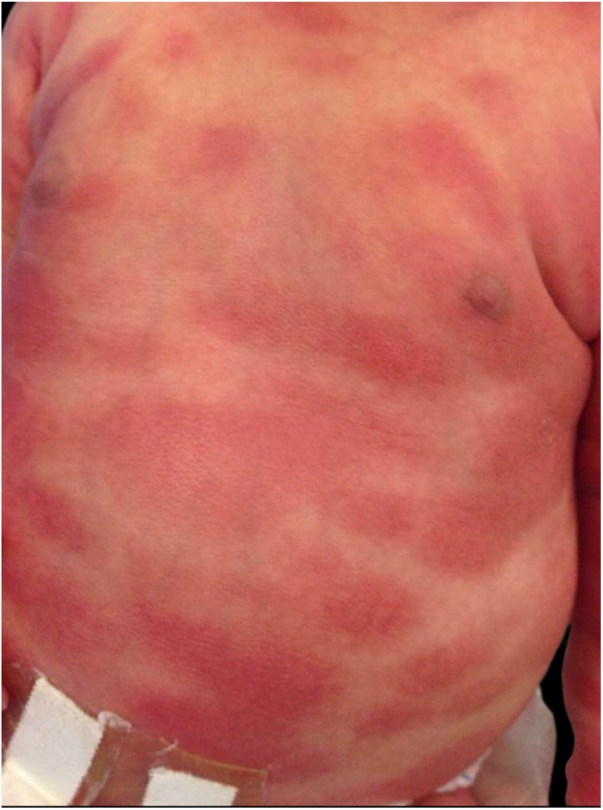
Erythematous plaques present at birth.

**Figure 2:**
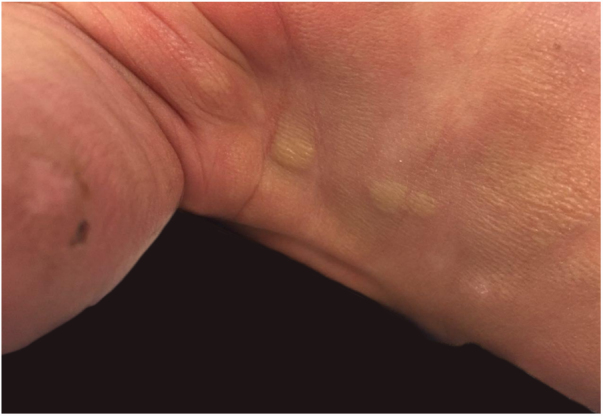
Erythematous brownish plaques on lower limb, topped by blisters at different stages of development (stray blisters, flaccid blisters and exulcerations).

At that moment, prednisone 1 mg/kg/day was introduced and a skin biopsy performed. The skin biopsy revealed numerous of diffuse mast cells cutaneous infiltrates stained in metachromically toluidine blue staining, confirming the diagnosis of mastocytosis (Fig. 3). Immunohistochemistry CD117 (c-kit) evidenced massive positivity of mast cells (Fig. 3). Serum tryptase levels were increased, and to rule out SM, a myelogram was performed, with a normal result.

**Figure 3:**
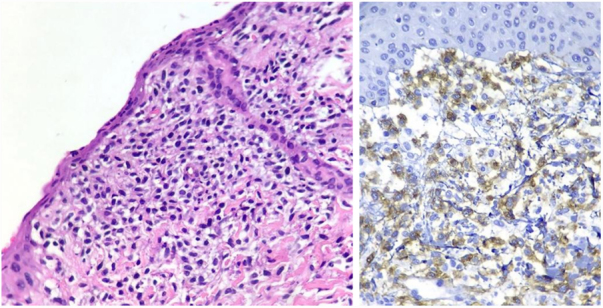
Left: numerous mast cells arranged diffusely in the dermis stained by Hematoxylin & eosin. Right: the CD117 immunohistochemical reaction (c-kit) is diffusely positive in mast cells.

In view of the clinical picture and additional tests, the child was diagnosed with bullous DCM and the progressive weaning of prednisone was made. Hydroxizine sedative dose was started, as well as guidance from the entire team to avoid possible triggers of mastocytosis.

At present, the child is in outpatient follow-up, with normal development, still remains with brownish lesions and some bullous in the whole tegument, but without signs of systemic disease.

Signs and symptoms found in mastocytosis are caused by excess mast cell degranulation, which can lead to cutaneous manifestations (such as itchiness, redness, edema), abdominal symptoms, respiratory symptoms, hypotension and even anaphylaxis.^1^ Cutaneous mastocytosis includes maculo-papular form, DCM and solitary mastocytoma.^1^, ^2^ Blisters can occur in all forms and are often associated with systemic involvement.^5^ Investigation of systemic disease is indicated when there are signs or symptoms consistent with systemic disease, or when the serum level of tryptase is above 20 ng/mL.^1^, ^5^ At that stage, directed imaging as well as bone marrow biopsy is crucial. The severity of the symptoms and skin infiltration tend to correlate with the serum level of tryptase, key enzyme in the metabolism of histamine and in the monitoring of mast cell activity.^1^, ^2^, ^3^ Our case did not show alterations of other organs beyond the skin, despite presenting high tryptase.

DCM is a rare condition that usually manifests with erythema, dermal thickening, skin folds accentuation, and edema with a typical leather or peau d’orange appearance.^2^, ^3^ The actual prognosis for DCM is debatable.^2^, ^3^, ^5^ According to some authors, most cases improve in months to years, while others believe that patients are at increased risk of developing SM or life-threatening events, such as hypotension or bronchospasm.^2^, ^4^

A review of DCM cases concluded that the majority were congenital and that even in cases of high tryptase the prognosis was good.^3^ Perhaps because elevated serum tryptase levels are correlated with systemic involvement in adults, but this association is not yet fully understood in children.^3^, ^4^ It seems to be more correlated to the extension of skin involved and the presence of symptoms.^4^ There is a higher risk of unfavorable evolution (anaphylactic shock and death) in children with neonatal onset, extensive and early blisters as well as vasodilation symptoms.^5^

Reliable prognostic criteria are still lacking to predict the risk of systemic involvement in DCM and maculo-papular mastocytosis. Most cases of DCM resolve spontaneously in childhood or adolescence, however these patients are at a greater risk of complications such as hypotension, anaphylaxis and diarrhea and they should be monitored regularly.

## Financial support

None declared.

## Authors’ contributions

Julia Marcon Cardoso: Approval of the final version of the manuscript; conception and planning of the study; elaboration and writing of the manuscript; critical review of the literature; critical review of the manuscript.

Camila Angelico S. Cabral: Conception and planning of the study; critical review of the literature;

Rute Facchini Lellis: Approval of the final version of the manuscript; obtaining, analysis, and interpretation of the data.

## Conflicts of interest

None declared.
